# Parental socioeconomic and lifestyle determinants of children’s secondhand smoke exposure in Hungary: analysis of European Health Interview Survey 2019 data

**DOI:** 10.3389/fpubh.2026.1834530

**Published:** 2026-08-06

**Authors:** Battamir Ulambayar, Attila Csaba Nagy

**Affiliations:** Department of Epidemiology, Faculty of Health Sciences, University of Debrecen, Debrecen, Hungary

**Keywords:** children, household exposure, Hungary, parental smoking, secondhand smoke, socioeconomic inequalities

## Abstract

**Background:**

Secondhand smoke (SHS) exposure remains a major public health concern, particularly for children who are highly vulnerable to the harmful effects of tobacco smoke. Despite comprehensive smoke-free policies in Hungary, children may still be exposed to SHS in private environments such as homes and outdoor settings. This study aimed to examine the prevalence and determinants of children’s exposure to SHS in Hungary.

**Methods:**

This cross-sectional study used nationally representative data from the European Health Interview Survey (EHIS) 2019 conducted in Hungary. Of 964 eligible parents living with at least one child aged 0–15 years, 191 with missing outcome or covariate data were excluded, yielding a final analytic sample of 773 parents. Children’s exposure was assessed from parental reports of exposure to conventional cigarette smoke and/or e-cigarette aerosol during the previous week. Three outcome variables were analyzed: indoor exposure, outdoor exposure, and overall exposure. Associations between parental demographic, socioeconomic, health, and lifestyle factors and children’s exposure were examined using Pearson’s chi-square tests and weighted multivariable logistic regression models.

**Results:**

Among the respondents, 8.8% reported indoor SHS exposure and 17.1% reported outdoor exposure among children, while 21.5% reported overall SHS exposure. Children’s exposure to SHS was significantly associated with several parental characteristics. Lower household income, non-registered marital status, poorer parental oral health, and active smoking were associated with higher odds of indoor SHS exposure. Outdoor exposure was associated with urbanization, poorer oral health, and parental smoking. Overall SHS exposure was higher among children of parents with lower income, non-registered marital status, poorer oral health, poorer mental health, and active smoking status.

**Conclusion:**

A considerable proportion of children in Hungary remain exposed to SHS. Socioeconomic disadvantage, parental smoking, and parental health status are important determinants of children’s SHS exposure. Targeted public health interventions focusing on smoking cessation and promoting smoke-free home environments are essential to reduce children’s exposure to SHS.

## 1 Introduction

Secondhand smoke (SHS) exposure remains a major public health concern worldwide, particularly for children (1). According to the World Health Organization, approximately 40% of children globally are regularly exposed to SHS, contributing to an estimated 65,000 deaths among children annually attributable to SHS exposure (2). Exposure to SHS has been associated with numerous adverse health outcomes in children, including respiratory infections, asthma, middle ear disease, impaired lung function, and increased risk of sudden infant death syndrome (3). Recent evidence based on the Global Burden of Disease 2019 study showed that approximately 6.9% of lower respiratory infection deaths among children under 5 years worldwide were attributable to SHS exposure in 2019, highlighting the substantial global health burden of SHS among children (4).

In European countries, despite strong tobacco control policies, exposure to SHS remains common, particularly in private and public environments where children spend substantial amounts of time, including homes, outdoor recreational areas, and shared community spaces (5). According to previous studies, a considerable proportion of children in Europe are still regularly exposed to tobacco smoke in household environments (6). Evidence also suggests that exposure may occur in outdoor settings; a multi-country European study detected airborne nicotine in over 40% of children’s playgrounds, indicating the presence of SHS even in outdoor play areas (7). In Hungary, although comprehensive smoke-free legislation has substantially reduced exposure to SHS in public places, smoking prevalence remains relatively high (8), and children may still be exposed to tobacco smoke in private and outdoor environments where smoking restrictions are less strictly enforced (9, 10).

Parental smoking behavior is therefore one of the most important determinants of children’s exposure to tobacco smoke. Previous studies have shown that children living with parents who smoke are significantly more likely to be exposed to SHS both indoors and outdoors (11). In addition to parental smoking behavior, several demographic and socioeconomic factors have been found to influence children’s exposure to SHS. Socioeconomic inequalities play a critical role in shaping tobacco-related health risks (12). Research from many countries has consistently demonstrated that children from socioeconomically disadvantaged households experience higher levels of SHS exposure. Lower parental education, lower household income, unemployment, and living in rural or disadvantaged areas have all been associated with increased exposure to tobacco smoke (13–15). These patterns may reflect differences in smoking prevalence, housing conditions, awareness of SHS risks, and the lack of household-level smoking restrictions and smoke-free home practices. These inequalities contribute to broader health disparities among children and represent an important challenge for public health policy.

Previous studies conducted in Hungary have examined aspects of smoking-related behaviors and health inequalities; however, the social and behavioral determinants of children’s SHS exposure in Hungary remain incompletely characterized. Specifically, prior work has not simultaneously examined indoor and outdoor exposure as distinct outcomes, has not explored the role of parental health-related factors such as oral health and mental health status as determinants of children’s exposure, and has not applied a comprehensive multivariable framework integrating socioeconomic, demographic, and lifestyle characteristics within a nationally representative sample. The present study addresses these gaps using data from the European Health Interview Survey (EHIS) 2019 in Hungary, the most current nationally representative dataset available, to provide an updated and comprehensive analysis of the prevalence and determinants of children’s SHS exposure across indoor, outdoor, and overall exposure environments.

## 2 Methods

### 2.1 Research design and data source

This study used data from the EHIS 2019 conducted in Hungary, a nationally representative cross-sectional survey designed to collect information on health status, health determinants, and healthcare utilization among the adult population. The survey was conducted by the Hungarian Central Statistical Office in 2019 using standardized questionnaires developed within the European Statistical System. The EHIS survey employs a multistage stratified probability sampling design to ensure national representativeness of the Hungarian population aged 15 years and older. Households were randomly selected, and one eligible respondent per household was interviewed using a structured questionnaire covering several thematic modules (16).

The full EHIS 2019 Hungary sample comprised 5,603 respondents. Of these, 964 were parents or guardians living with at least one child aged 0–15 years and were considered eligible for inclusion in the present study. Of the 964 eligible respondents, 191 (19.8%) were excluded due to missing values on the SHS exposure outcome variables or on key socioeconomic and lifestyle covariates, resulting in a final analytic sample of 773 parents. The majority of these exclusions were attributable to missing responses to the EHIS Passive Smoking module, from which the children’s SHS exposure outcome variables were derived, whereas the remaining socioeconomic and lifestyle covariates were largely complete. To assess potential selection bias introduced by these exclusions, the demographic and socioeconomic characteristics of the excluded respondents were compared with those of the included analytic sample. No statistically significant differences were observed between the two groups with respect to age, sex, educational attainment, or degree of urbanization (all *p* > 0.05), suggesting that the exclusions were unlikely to have introduced systematic bias into the study findings.

### 2.2 Variables

The primary outcomes of interest were children’s exposure to SHS. These variables were derived from the Passive Smoking module of the EHIS questionnaire (Chapter 9), which separately assessed children’s exposure to conventional cigarette smoke and e-cigarette aerosol in their presence across different environments during the previous week. Although e-cigarette aerosol and combustible cigarette smoke differ in their toxicological profiles and may have distinct socioeconomic determinants, these exposure categories were combined in the present analysis because standalone e-cigarette aerosol exposure was rare in the study population, reflecting the comparatively lower prevalence of vaping in Hungary relative to traditional smoking at the time of the survey. This decision prioritizes analytical feasibility over granularity, and future studies with larger vaping-exposed subgroups should examine these exposure types separately. The study primarily aimed to examine parental smoking/vaping-related behaviors and children’s passive exposure within household and surrounding environments. Based on these questionnaire items, three outcome variables were constructed: indoor SHS exposure, outdoor SHS exposure, and overall SHS exposure. Indoor SHS exposure refers to children being exposed inside the home or another closed place during the previous week, while outdoor SHS exposure refers to exposure occurring in outdoor environments. Overall SHS exposure was defined as exposure occurring in either indoor or outdoor environments on more than one occasion during the previous week. This variable was intended to capture the presence of children’s passive exposure across environments rather than cumulative exposure intensity or simultaneous indoor and outdoor exposure.

Independent variables included parental demographic, socioeconomic, health, and lifestyle characteristics obtained from the EHIS 2019 questionnaire. Several original EHIS variables were recategorized for statistical analysis to improve interpretability and ensure adequate subgroup sample sizes. Age was categorized into 18–34 years and 35–64 years. Educational attainment was grouped into primary, secondary, and higher education. Income quintiles were predefined by EHIS using equivalized household income distributions at the national level. The first quintile represented the lowest household income group, while the fifth quintile represented the highest household income group. Marital status was categorized as registered (legally married), not registered (cohabiting but not legally married), and single parent. Employment status was categorized as active (currently employed) and inactive (including unemployed, retired, students, homemakers, or other individuals not currently employed). Self-perceived health status and oral health status were categorized as very good/good, satisfactory, and bad/very bad. Mental health status was assessed using the WHO-5 Well-Being Index and categorized as better or poor mental health according to established cut-off criteria. Smoking status was categorized as active smoking, former smoking, and non-smoking. Variables related to smoking duration, age at smoking initiation, cigarette consumption, and alcohol use were also recategorized into broader groups for analysis.

### 2.3 Statistical analysis

Descriptive statistics were used to summarize the characteristics of the study population. The prevalence of indoor, outdoor, and overall SHS exposure among children was calculated and presented as frequencies and percentages. Associations between parental demographic, socioeconomic, health, and lifestyle characteristics and children’s SHS exposure were first examined using bivariate analyses. Pearson’s chi-squared tests were applied to assess statistical differences between groups. To identify independent associations of children’s SHS exposure, multivariable logistic regression models were performed. Separate models were constructed for indoor SHS exposure, outdoor SHS exposure, and overall SHS exposure. Adjusted odds ratios (ORs) with 95% confidence intervals (CIs) were calculated to estimate the strength of associations between parental characteristics and children’s exposure to SHS. Variables excluded from the multivariable models due to collinearity included smoking duration, age at smoking initiation, and cigarette consumption, as these variables were strongly correlated with smoking status. Health problems limiting daily activities were excluded because of multicollinearity with general health status. Multicollinearity among the retained variables was formally assessed using the Variance Inflation Factor (VIF). All VIF values were below 5 (mean VIF = 1.78, maximum VIF = 3.56), indicating no multicollinearity concern in the all three models. To examine whether the association between degree of urbanization and overall SHS exposure varied across household income levels, an interaction term between these two variables (urbanization × household income) was introduced into the overall SHS exposure model, and predicted probabilities were estimated using marginal effects with 95% confidence intervals. Sampling weights provided by the Hungarian Central Statistical Office were applied throughout all descriptive, bivariate analyses and multivariable logistic regression models, and all analyses accounted for the complex survey design of the EHIS 2019 using the svy commands in Stata IC 18.0 (StataCorp, College Station, TX, USA) (17) to obtain nationally representative estimates.

## 3 Results

In total, 773 parents with children aged 0–15 years were included in the analysis. Among them, 62 (8.8%) reported indoor SHS exposure, 132 (17.1%) reported outdoor SHS exposure, and 166 (21.5%) reported overall SHS exposure among their children during the previous week.

Bivariate analyses demonstrated that children’s SHS exposure was significantly associated with several parental demographic and socioeconomic characteristics (Table 1). Indoor SHS exposure was more common among parents with lower educational attainment, lower household income, inactive employment status, rural residence, and non-registered marital status. Similar patterns were observed for overall SHS exposure, which was also higher among younger parents and single-parent households. Outdoor SHS exposure was significantly associated with parental age, marital status, and degree of urbanization, with higher exposure observed among younger parents and those living in rural areas. Overall, children living in socioeconomically disadvantaged households consistently showed higher levels of SHS exposure across exposure environments.

**Table 1:** Parents’ demographic and socioeconomic factors associated with SHS among children.

Variables (Parents)		Indoor SHS among child(ren)		*p* value*	Outdoor SHS among child(ren)		*p* value*	Overall SHS among child(ren)		*p* value*
		No (*n*, %)	Yes (*n*, %)		No (*n*, %)	Yes (*n*, %)		No (*n*, %)	Yes (*n*, %)	
Gender	Male	310 (92.3)	26 (7.7)	0.362	286 (85.1)	50 (14.9)	0.155	271 (80.6)	65 (19.4)	0.206
	Female	395 (90.4)	42 (9.6)		355 (81.2)	82 (18.8)		336 (76.9)	101 (23.1)	
Age group	18–34 years old	193 (87.7)	27 (12.3)	0.089	169 (76.8)	51 (23.2)	**0.014**	159 (72.3)	61 (27.7)	**0.02**
	35–64 years old	509 (92.5)	41 (7.5)		469 (85.3)	81 (14.7)		445 (80.9)	105 (19.1)	
	65 and older	3 (100.0)	0 (0)		3 (100.0)	0 (0)		3 (100.0)	0 (0)	
Educational attainment	Primary	98 (73.7)	35 (26.3)	**<0.001**	104 (78.2)	29 (21.8)	0.082	86 (64.7)	47 (35.3)	**<0.001**
	Secondary	382 (93.4)	27 (6.6)		336 (82.2)	73 (17.8)		322 (78.7)	87 (21.3)	
	Higher	225 (97.4)	6 (2.6)		201 (87.0)	30 (13.0)		199 (86.1)	32 (13.9)	
Self-reported income levels	First quintile (lowest)	143 (77.7)	41 (22.3)	**<0.001**	136 (73.9)	48 (26.1)	**0.005**	116 (63.0)	68 (37.0)	**<0.001**
	Second quintile	124 (92.5)	10 (7.5)		114 (85.1)	20 (14.9)		108 (80.6)	26 (19.4)	
	Third quintile	142 (95.9)	6 (4.1)		130 (87.8)	18 (12.2)		126 (85.1)	22 (14.9)	
	Fourth quintile	175 (95.1)	9 (4.9)		155 (84.2)	29 (15.8)		152 (82.6)	32 (17.4)	
	Fifth quintile (highest)	121 (98.4)	2 (1.6)		106 (86.2)	17 (13.8)		105 (85.4)	18 (14.6)	
Employment status	Active	539 (93.3)	39 (6.7)	**0.001**	488 (84.4)	90 (15.6)	0.056	470 (81.3)	108 (18.7)	**0.001**
	Inactive	166 (85.1)	29 (14.9)		153 (78.5)	42 (21.5)		137 (70.3)	58 (29.7)	
Degree of urbanization	City	211 (96.4)	8 (3.6)	**<0.001**	182 (83.1)	37 (16.9)	**<0.001**	180 (82.2)	39 (17.8)	**<0.001**
	Town/suburb	247 (94.7)	14 (5.4)		237 (90.8)	24 (9.2)		228 (87.4)	33 (12.6)	
	Rural area	247 (84.3)	46 (15.7)		222 (75.8)	71 (24.2)		199 (67.9)	94 (32.1)	
Marital status	Registered	504 (94.2)	31 (5.8)	**<0.001**	458 (85.6)	77 (14.4)	**0.005**	441 (82.4)	94 (17.6)	**<0.001**
	Not registered	128 (81.5)	29 (18.5)		122 (77.7)	35 (22.3)		106 (67.5)	51 (32.5)	
	Single parent	63 (90.0)	7 (10.0)		51 (72.9)	19 (27.1)		51 (72.9)0	19 (27.1)	

*Bold values indicate statistical significance (*p* < 0.05) based on Pearson’s chi-squared test.

Several parental health and lifestyle characteristics were also significantly associated with children’s SHS exposure (Table 2). Indoor SHS exposure was associated with poorer self-perceived health, poorer oral health, active smoking, earlier smoking initiation, longer smoking duration, and higher daily cigarette consumption. Outdoor exposure was significantly associated with activity-limiting health problems, poorer mental health, poorer oral health, and smoking status. Similarly, overall SHS exposure was more common among parents reporting poorer self-perceived health, poorer oral health, poor mental health, and active smoking behavior. Across all exposure categories, children of active smokers showed substantially higher levels of SHS exposure compared with children of former smokers or non-smoking parents.

**Table 2:** Parents’ health and lifestyle factors associated with SHS among children.

Variables (Parents)		Indoor SHS among child(ren)		*p* value*	Outdoor SHS among child(ren)		*p* value*	Overall SHS among child(ren)		*p* value*
		No (*n*, %)	Yes (*n*, %)		No (*n*, %)	Yes (*n*, %)		No (*n*, %)	Yes (*n*, %)	
Self-perceived health status	Very good or good	563 (92.3)	47 (7.7)	**0.006**	512 (83.9)	98 (16.1)	0.333	492 (80.7)	118 (19.3)	**0.015**
	Satisfactory	124 (89.2)	15 (10.8)		110 (79.1)	29 (20.9)		99 (71.2)	40 (28.8)	
	Bad or very bad	17 (73.9)	6 (26.1)		18 (78.3)	5 (21.7)		15 (65.2)	8 (34.8)	
Long-term illness	Yes	208 (91.6)	19 (8.4)	0.761	186 (81.9)	41 (18.1)	0.536	179 (78.8)	48 (21.2)	0.997
	No	482 (90.9)	48 (9.1)		444 (83.8)	86 (16.2)		418 (78.9)	112 (21.1)	
Health problems that limit daily activities	Yes	83 (88.3)	11 (11.7)	0.295	68 (72.3)	26 (27.7)	**0.003**	63 (67.0)	31 (33.0)	**0.004**
	No	619 (91.6)	57 (8.4)		571 (84.5)	105 (15.5)		542 (80.2)	134 (19.8)	
Self-rated oral health status	Very good or good	398 (95.0)	21 (5.0)	**<0.001**	370 (88.3)	49 (11.7)	**<0.001**	361 (86.2)	58 (13.8)	**<0.001**
	Satisfactory	208 (91.2)	20 (8.8)		179 (78.5)	49 (21.5)		169 (74.1)	59 (25.9)	
	Bad or very bad	98 (78.4)	27 (21.6)		91 (72.8)	34 (27.2)		76 (60.8)	49 (39.2)	
Mental health status (WHO-5)	Better	580 (91.9)	51 (8.1)	0.139	533 (84.5)	98 (15.5)	**0.016**	508 (80.5)	123 (19.5)	**<0.001**
	Poor	125 (88.0)	17 (12.0)		108 (76.1)	34 (23.9)		99 (69.7)	43 (30.3)	
Smoking status	Active smoking	215 (82.7)	45 (17.3)	**<0.001**	197 (75.8)	63 (24.2)	**<0.001**	173 (66.5)	87 (33.5)	**<0.001**
	Former smoking	120 (98.4)	2 (1.6)		99 (81.2)	23 (18.8)		98 (80.3)	24 (19.7)	
	No smoking	369 (94.6)	21 (5.4)		344 (88.2)	46 (11.8)		335 (85.9)	55 (14.1)	
Age of beginning smoking	Before turning 16	82 (81.2)	19 (18.8)	**0.02**	73 (72.3)	28 (27.7)	0.139	63 (62.4)	38 (37.6)	**0.026**
	16 or later	254 (90.1)	28 (9.9)		224 (79.4)	58 (20.6)		209 (74.1)	73 (25.9)	
Years of smoking	Up to 10 years	128 (91.4)	12 (8.6)	**0.027**	111 (79.3)	29 (20.7)	0.690	106 (75.7)	34 (24.3)	0.228
	10–20 years	110 (88.7)	14 (11.3)		93 (75.0)	31 (25.0)		85 (68.6)	39 (31.4)	
	More than 20 years	70 (79.5)	18 (20.5)		69 (78.4)	19 (21.6)		58 (65.9)	30 (34.1)	
Average daily cigarette consumption	Up to 10 cigarettes	57 (87.7)	8 (12.3)	**0.003**	52 (80.0)	13 (20.0)	0.376	47 (72.3)	18 (27.7)	0.064
	10–20 cigarettes	91 (85.1)	16 (14.9)		78 (72.9)	29 (27.1)		71 (66.4)	36 (33.6)	
	More than 30 cigarettes	14 (58.3)	10 (41.7)		16 (66.7)	8 (33.3)		11 (45.8)	13 (54.2)	
Alcohol consumptions	Heavy	29 (80.6)	7 (19.4)	0.066	25 (69.4)	11 (30.6)	0.169	23 (63.9)	13 (36.1)	0.163
	Moderate	104 (92.9)	8 (7.1)		93 (83.0)	19 (17.0)		89 (79.5)	23 (20.5)	
	Rare	373 (92.6)	30 (7.4)		339 (84.1)	64 (15.9)		322 (79.9)	81 (20.1)	
	No alcohol	195 (89.5)	23 (10.5)		181 (83.0)	37 (17.0)		170 (78.0)	48 (22.0)	
Physical effort at work/main activity	Mostly light effort	98 (92.5)	8 (7.5)	0.118	89 (83.9)	17 (16.1)	0.900	85 (80.2)	21 (19.8)	0.483
	Mostly heavy physical work	59 (85.5)	10 (14.5)		60 (87.0)	9 (13.0)		53 (76.8)	16 (23.2)	
	Mostly walks or moderate physical work	303 (90.2)	33 (9.8)		276 (82.1)	60 (17.9)		260 (77.4)	76 (22.6)	
	Mostly sitting	237 (93.3)	17 (6.7)		212 (83.5)	42 (16.5)		204 (80.3)	50 (19.7)	

*Bold values indicate statistical significance (*p* < 0.05) based on Pearson’s chi-squared test.

Multivariable logistic regression analyses identified several independent predictors of children’s SHS exposure (Table 3). Lower household income remained significantly associated with increased odds of indoor and overall SHS exposure. Compared with the lowest income quintile, parents in the second (OR = 0.37, 95% CI: 0.16–0.84), third income quintiles (OR = 0.32, 95% CI: 0.11–0.90), and fifth income quintiles (OR = 0.19, 95% CI: 0.03–0.97) had significantly lower odds of indoor SHS exposure, while parents in the second (OR = 0.47, 95% CI: 0.26–0.84) and third income quintiles (OR = 0.45, 95% CI: 0.23–0.86) also had lower odds of overall SHS exposure. Marital status also remained significant, as children of parents who were not legally married had higher odds of indoor (OR = 2.09, 95% CI: 1.11–3.92) and overall SHS exposure (OR = 1.58, 95% CI: 1.01–2.51) compared with children of legally married parents. Degree of urbanization was independently associated with outdoor and overall exposure, with children living in towns or suburban areas showing lower odds of outdoor (OR = 0.36, 95% CI: 0.20–0.67) and overall SHS exposure (OR = 0.46, 95% CI: 0.26–0.81) compared with those living in cities.

**Table 3:** Multivariable logistic regression analysis of factors associated with indoor, outdoor, and overall SHS exposure among children.

Variables (Parents)		Indoor SHS among child(ren)		*p* value*	Outdoor SHS among child(ren)		*p* value*	Overall SHS among child(ren)		*p* value*
		OR	95% CI		OR	95% CI		OR	95% CI	
Sex	Male (Ref)									
	Female	1.52	0.81–2.85	0.183	1.53	0.97–2.41	0.064	1.49	0.97–2.29	0.064
Age	18–34 years old (Ref)									
	35–64 years old	1.42	0.75–2.69	0.277	0.72	0.45–1.14	0.166	1.01	0.65–1.57	0.945
Educational attainment	Primary (Ref)									
	Secondary	0.57	0.29–1.14	0.117	1.63	0.89–2.99	0.108	1.24	0.71–2.14	0.440
	Higher	0.45	0.13–1.58	0.218	1.56	0.67–3.64	0.298	1.19	0.54–2.62	0.648
Self-reported income levels	First quintile (Ref)									
	Second quintile	0.37	0.16–0.84	**0.017**	0.55	0.29–1.05	0.072	0.47	0.26–0.84	**0.012**
	Third quintile	0.32	0.11–0.90	**0.029**	0.48	0.23–0.97	**0.043**	0.45	0.23–0.86	**0.017**
	Fourth quintile	0.52	0.19–1.44	0.215	0.86	0.43–1.73	0.688	0.72	0.37–1.38	0.325
	Fifth quintile	0.19	0.03–0.97	**0.047**	0.75	0.33–1.72	0.504	0.60	0.27–1.31	0.202
Employment status	Active (Ref)									
	Inactive	0.95	0.48–1.89	0.897	0.91	0.54–1.53	0.748	1.08	0.66–1.75	0.747
Degree of urbanization	City (Ref)									
	Town/suburb	0.79	0.30–2.09	0.642	0.36	0.20–0.67	**0.001**	0.46	0.26–0.81	**0.008**
	Rural area	1.84	0.75–4.49	0.180	1.29	0.76–2.20	0.342	1.53	0.92–2.56	0.099
Marital status	Registered (Ref)									
	Not registered	2.09	1.11–3.92	**0.021**	1.29	0.78–2.13	0.319	1.58	1.01–2.51	**0.044**
	Single parent	1.33	0.51–3.7	0.549	2.05	1.09–3.78	**0.026**	1.44	0.76–2.73	0.252
Self-rated oral health status	Very good or good (Ref)									
	Satisfactory	1.25	0.61–2.57	0.539	1.89	1.17–3.04	**0.009**	1.89	1.20–2.97	**0.005**
	Bad or very bad	2.21	1.04–4.71	**0.038**	2.14	1.20–3.84	**0.01**	2.59	1.51–4.43	**<0.001**
Mental health status	Poor (Ref)									
	Better	0.81	0.41–1.61	**0.565**	0.65	0.40–1.05	**0.083**	0.61	0.38–0.97	**0.039**
Smoking status	Active smoking (Ref)									
	Former smoking	0.13	0.03–0.58	**0.007**	0.65	0.40–1.05	0.083	0.64	0.36–1.13	0.128
	No smoking	0.46	0.24–0.88	**0.019**	0.48	0.29–0.77	**0.003**	0.41	0.26–0.65	**<0.001**

*Bold values indicate statistical significance (*p* < 0.05) Odds ratios (OR) are adjusted for variables in the model.

Parental oral health status was independently associated with indoor, outdoor, and overall SHS exposure. Parents reporting bad or very bad oral health had significantly higher odds of indoor (OR = 2.21, 95% CI: 1.04–4.71), outdoor (OR = 2.14, 95% CI: 1.20–3.84), and overall SHS exposure (OR = 2.59, 95% CI: 1.51–4.43). Better parental mental health was associated with lower odds of overall SHS exposure (OR = 0.61, 95% CI: 0.38–0.97). In addition, parental smoking status remained one of the strongest predictors of exposure. Children of non-smoking parents had significantly lower odds of indoor (OR = 0.46, 95% CI: 0.24–0.88), outdoor (OR = 0.48, 95% CI: 0.29–0.77), and overall SHS exposure (OR = 0.41, 95% CI: 0.26–0.65) compared with children of active smokers, while children of former smokers also showed reduced odds of indoor exposure (OR = 0.13, 95% CI: 0.03–0.58).

To further examine whether the association between degree of urbanization and overall SHS exposure varied across household income levels, an interaction term between urbanization and household income was introduced into the overall SHS exposure model. The interaction was statistically significant for several combinations (Table 4). Among rural residents, the predicted probability of overall SHS exposure was highest in the lowest income quintile (38.9%) and the fourth income quintile (38.4%), while middle-income rural households showed substantially lower predicted probabilities (3rd quintile: 16.5%). Significant interaction terms were observed for rural × second quintile (OR = 0.116, 95% CI: 0.017–0.795, *p* = 0.028) and rural × third quintile (OR = 0.134, 95% CI: 0.019–0.949, *p* = 0.044), indicating that the rural disadvantage in SHS exposure was significantly attenuated among middle-income households. Among town and suburban residents, a significant interaction was found for town/suburb × second quintile (OR = 0.032, 95% CI: 0.003–0.348, *p* = 0.005). Notably, city residents in the second income quintile showed a relatively high predicted probability of exposure (29.3%), suggesting that urban poverty may also concentrate SHS risk independently of residential setting.

**Table 4:** Predicted probabilities of overall secondhand smoke exposure among children by degree of urbanization and household income quintile: marginal effects from the interaction model.

Income quintile	Urbanization	Predicted probability	95% CI Lower	95% CI Upper	*p*-value*
(A) Predicted probabilities of overall SHS exposure by urbanization and income (marginal effects)					
1st (lowest)	City	0.110	−0.031	0.251	0.127
	Town/Suburb	0.206	0.104	0.308	**<0.001**
	Rural	0.389	0.290	0.488	**<0.001**
2nd	City	0.293	0.122	0.464	**0.001**
	Town/Suburb	0.034	−0.012	0.080	0.148
	Rural	0.232	0.130	0.335	<0.001
3rd	City	0.192	0.073	0.312	**0.002**
	Town/Suburb	0.124	0.034	0.214	**0.007**
	Rural	0.165	0.065	0.265	**0.001**
4th	City	0.179	0.080	0.278	**<0.001**
	Town/Suburb	0.117	0.031	0.203	**0.008**
	Rural	0.384	0.250	0.518	**<0.001**
5th (highest)	City	0.223	0.111	0.334	**<0.001**
	Town/Suburb	0.106	0.009	0.203	**0.032**
	Rural	0.175	−0.032	0.381	0.097

Interaction term	Odds ratio	95% CI Lower	95% CI Upper	*p*-value
(B) Interaction term odds ratios (Urbanization × Income, Reference: City × 1st Quintile)				
Town/Suburb × 2nd quintile	0.032	0.003	0.348	**0.005**
Town/Suburb × 3rd quintile	0.255	0.032	2.010	0.195
Town/Suburb × 4th quintile	0.263	0.035	1.963	0.193
Town/Suburb × 5th quintile	0.172	0.021	1.393	0.099
Rural × 2nd quintile	0.116	0.017	0.795	**0.028**
Rural × 3rd quintile	0.134	0.019	0.949	**0.044**
Rural × 4th quintile	0.528	0.084	3.324	0.497
Rural × 5th quintile	0.118	0.012	1.189	0.070

*Bold values indicate statistical significance (*p* < 0.05). Predicted probabilities derived from marginal effects following multivariable logistic regression. Model adjusted for sex, age, educational attainment, employment status, marital status, smoking status, oral health status, and mental health status (WHO-5).

## 4 Discussion

This study examined the prevalence and determinants of children’s SHS exposure in Hungary using nationally representative data from the EHIS 2019. The findings indicate that a considerable proportion of children are still exposed to SHS, both indoors and outdoors. Children’s exposure was strongly associated with several parental characteristics, particularly socioeconomic status, smoking behavior, and health-related factors. Higher exposure was observed among children of parents with lower education and income levels, those living in rural areas, and those with non-registered marital status. In addition, parental smoking behavior and poorer oral and mental health were important factors associated with increased exposure. These findings highlight the persistent socioeconomic and behavioral inequalities in children’s exposure to SHS in Hungary.

One of the most important determinants of children’s SHS exposure identified in this study was parental self-reported income level. This finding is consistent with evidence from studies conducted in various regions of the world, which have consistently shown that socioeconomic disadvantage is strongly associated with higher levels of SHS exposure among children (9). This disparity is repeatedly linked to parental smoking (18), lower educational attainment (19), higher household tobacco use (20), limited adoption of smoke-free home rules, and broader social-contextual factors that shape smoking norms and environments (21). These findings highlight the persistent socioeconomic gradient in tobacco-related health risks and underscore the need for targeted public health interventions aimed at reducing SHS exposure among socioeconomically disadvantaged families.

The degree of urbanization was another factor associated with children’s exposure to SHS in the present study. Similar patterns have been reported in previous research, suggesting that SHS exposure among children often varies across urban and rural settings. Higher exposure in non-urban areas may be partly explained by higher smoking prevalence, weaker enforcement of smoke-free rules, and lower adoption of smoke-free homes in rural populations (22–24). At the same time, evidence indicates that urban environments may also be associated with SHS exposure due to housing density and the presence of multiunit dwellings, where smoke can spread between apartments (25, 26). Therefore, the relationship between urbanization and children’s SHS exposure is complex and may depend on contextual factors such as housing conditions, social norms around smoking, and the enforcement of smoke-free policies (27, 28). These findings suggest that strategies aimed at reducing children’s SHS exposure should consider urban–rural differences and address environmental and social determinants of tobacco smoke exposure across different residential settings.

The interaction analysis between urbanization and household income revealed that the association between residential setting and children’s overall SHS exposure is not uniform across income levels, but rather depends on the interplay between place of residence and socioeconomic resources. Rural disadvantage in SHS exposure was most pronounced among the lowest and fourth income quintile households, while middle-income rural families showed predicted probabilities comparable to or lower than their urban counterparts. This pattern suggests that sufficient household income may buffer the rural disadvantage in SHS exposure, possibly through greater capacity to adopt and enforce smoke-free home rules, access smoking cessation support, or reside in better-quality housing that limits smoke infiltration. Conversely, the relatively high predicted probability of SHS exposure observed among second quintile city residents highlights that urban residence does not inherently protect children from SHS exposure when household resources remain limited. These findings underscore the importance of considering the joint effects of socioeconomic and environmental factors when designing targeted public health interventions, as neither urbanization nor income alone fully captures the heterogeneity of children’s SHS exposure risk across residential settings. An unexpected finding was the relatively high predicted probability of overall SHS exposure among rural households in the fourth income quintile, which was comparable to that observed in the lowest income quintile. Although this subgroup was not characterized by a particularly small sample size, the non-linear pattern should be interpreted cautiously, as interaction estimates may be influenced by residual heterogeneity or sampling variability. Given the exploratory nature of the interaction analysis, this finding should be considered hypothesis-generating and warrants confirmation in future studies.

Parental marital status was also significantly associated with children’s exposure to SHS in this study. Specifically, children living in households where parents were not registered as married had higher odds of indoor SHS exposure and a greater likelihood of overall exposure compared with those living with registered married parents. Similarly, children living with single parents showed higher levels of outdoor exposure. Previous research indicates that marital status is closely linked to adult health behaviors, including smoking, which is a major determinant of children’s SHS exposure. For example, Ramsey et al. reported that detailed marital status categories were associated with current cigarette smoking in the United States, with differences observed across racial and ethnic groups (29). This suggests that unmarried status may be linked to higher or more variable smoking prevalence, potentially increasing children’s exposure to SHS in the household environment. In addition, studies examining cancer outcomes and mortality patterns have shown that marital status is associated with health behaviors and treatment decisions, often reflecting broader differences in social support and stress levels (30, 31). Taken together, these findings suggest that family structure may be associated with smoking-related behaviors and the adoption of smoke-free home environments, and with children’s risk of SHS exposure.

The variables of health status, including the parental oral health and mental health status, were also significantly associated with children’s exposure to SHS smoke in this study. Children whose parents reported poorer oral health had significantly higher odds of indoor, outdoor, and overall SHS exposure compared with those whose parents reported good oral health. This relationship may reflect broader patterns of health behavior, as poor oral health is frequently associated with unhealthy lifestyle factors, including tobacco use and lower engagement in preventive health practices. Previous studies have demonstrated strong links between smoking and adverse oral health outcomes, including periodontal disease and tooth loss, indicating that tobacco use is strongly associated with poor oral health status (32, 33). In addition, oral health has been described as part of a broader framework of shared behavioral risk factors, where smoking, diet, and other lifestyle behaviors cluster together and influence overall health outcomes (34). Although oral health status remained independently associated with children’s SHS exposure after adjustment for measured socioeconomic characteristics, it may represent a marker of broader socioeconomic disadvantage and shared behavioral risk factors rather than a distinct causal behavioral determinant. Consistent with the Common Risk Factor Approach (35), poor oral health and tobacco exposure are influenced by common upstream social and behavioral determinants, including socioeconomic position, health literacy, access to preventive care, and health-related behaviors (36). Consequently, residual socioeconomic confounding cannot be excluded, and parental oral health may best be viewed as a downstream indicator of health inequalities rather than an independent driver of children’s SHS exposure.

Likewise, extensive evidence shows strong associations between smoking and mental health conditions, including depression, anxiety, and psychological distress, with affected individuals more likely to initiate or maintain smoking behaviors (37, 38). Psychological distress has been associated with smoking as a coping behavior, which may in turn be linked to higher smoking frequency in the household environment and reduce the ability to maintain smoke-free home rules. Consequently, poorer parental oral and mental health may be indirectly associated with increased children’s exposure to SHS through higher smoking prevalence and weaker enforcement of smoke-free environments.

Finally, parental smoking status was one of the strongest determinants of children’s exposure to SHS in this study. These findings are consistent with a large body of evidence demonstrating that parental smoking is the strongest predictor of children’s exposure to SHS, particularly within the home environment (39). Previous studies have shown that children living with smoking parents are significantly more likely to be exposed to tobacco smoke both indoors and outdoors, as smoking in homes, cars, and shared family environments is associated with higher exposure levels (40, 41). Systematic reviews have similarly identified parental smoking behavior as the most important predictor of children’s SHS exposure at home, highlighting the strong association between household smoking practices and children’s environmental health risks. These findings suggest that household smoking practices and the adoption of smoke-free home rules are closely linked to parental smoking status, underscoring the importance of smoking cessation as the most direct modifiable factor for reducing children’s SHS exposure.

The findings of the present study carry specific implications for tobacco control policy in Hungary. Although Hungary has implemented comprehensive smoke-free legislation since 2012 (42), and confined tobacco retail exclusively to National Tobacco Shops since 2013, a measure associated with short-term reductions in youth smoking prevalence (43), these policies primarily target public spaces and retail access, leaving private environments such as homes and outdoor residential areas largely unregulated. The persistent rural disadvantage in children’s SHS exposure observed in this study suggests that enforcement of smoke-free norms and access to cessation support remain disproportionately limited in non-urban settings, where smoking prevalence is higher and social norms around smoking are less restrictive. Targeted outreach through primary care and community health services in rural areas, combined with the promotion of voluntary smoke-free home pledges, could help address this gap. The association between poor parental mental health and children’s SHS exposure points to the need for integrated approaches that combine smoking cessation support with mental health services, particularly given the well-documented co-occurrence of smoking and psychological distress. In this regard, Hungary’s primary care system could serve as an important entry point for identifying families at elevated risk and delivering brief motivational interventions. Similarly, the higher SHS exposure observed among children of non-registered and single parents highlights the need for family-centered interventions that do not assume a traditional household structure, and that address the social isolation and reduced support networks frequently associated with non-standard family arrangements.

This study has several strengths and limitations that should be considered when interpreting the findings. A major strength is the use of nationally representative data from the EHIS 2019, which provides comprehensive information on demographic, socioeconomic, health, and lifestyle characteristics of parents. The relatively large sample of households with children also allowed for the examination of multiple potential determinants of children’s SHS exposure. In addition, the study considered different environments of exposure, including indoor, outdoor, and overall SHS exposure, providing a broader understanding of children’s exposure patterns.

However, several limitations should also be acknowledged. First, the cross-sectional design of the EHIS survey does not allow causal relationships to be established between parental characteristics and children’s SHS exposure. Second, SHS exposure and parental behaviors were based on self-reported data, which may be subject to recall bias or social desirability bias, particularly for smoking-related behaviors. In this regard, the relatively low prevalence of indoor SHS exposure (8.8%) should be interpreted with caution, as it may partly reflect underreporting driven by social desirability bias; given the increasing anti-smoking norms in Hungary and broader Europe, parents may be reluctant to report exposing their children to tobacco smoke indoors, potentially leading to underestimation of true indoor exposure rates. Third, the survey did not include objective biomarkers of tobacco smoke exposure, such as cotinine measurements or air nicotine monitoring, which would provide a more precise and less bias-prone assessment of children’s exposure levels. In addition, although conventional cigarette smoke exposure and e-cigarette aerosol exposure were assessed separately in the EHIS questionnaire, these variables were combined in the present analysis because exposure to e-cigarette aerosol alone was uncommon in the study population. Therefore, the findings primarily reflect conventional cigarette smoke exposure. The present study mainly focused on parental smoking/vaping-related behaviors and children’s passive exposure environments rather than the specific toxicological differences between cigarette smoke and e-cigarette aerosol. Future studies should evaluate these exposure types separately. Furthermore, the dataset did not include information on housing characteristics or living arrangements, such as residence in apartments versus detached houses. This may be relevant because previous studies have shown that children living in multi-unit housing may experience higher SHS exposure due to smoke infiltration from neighboring units. Finally, some potentially relevant household factors, such as the presence of strict smoke-free home rules or the smoking behavior of other household members, were not available in the dataset. Despite these limitations, the study provides valuable insights into the socioeconomic and behavioral determinants of children’s SHS exposure in Hungary and highlights important areas for targeted public health interventions.

## 5 Conclusion

SHS exposure remains a significant public health concern among children in Hungary. This study found that a considerable proportion of children were exposed to SHS, with exposure strongly associated with several parental socioeconomic and demographic factors. Children living in households with lower income, lower educational attainment, and economically inactive parents were more likely to be exposed to SHS. Higher exposure was also observed among children living in rural areas and in households with non-registered or single-parent marital status, highlighting the presence of socioeconomic inequalities in children’s exposure to SHS.

Parental health and lifestyle characteristics also played an important role. Poorer parental oral health and smoking behavior were among the strongest predictors of children’s exposure, while children of non-smoking parents had significantly lower odds of exposure. These findings suggest that reducing children’s exposure to SHS requires targeted interventions focusing on parental smoking cessation and the promotion of smoke-free home environments, particularly among socioeconomically disadvantaged populations. Strengthening tobacco control policies and increasing awareness of the health risks associated with SHS may help reduce exposure and improve child health outcomes.

## Data Availability

The data presented in this study are available upon request from Hungarian Central Statistical Office who performed and supervised the data collection. Requests to access these datasets should be directed to Hungarian Central Statistical Office, https://www.ksh.hu/?lang=en.
